# A Systematic Review and Meta-Regression Analysis of Lung Cancer Risk and Inorganic Arsenic in Drinking Water

**DOI:** 10.3390/ijerph121214990

**Published:** 2015-12-07

**Authors:** Steven H. Lamm, Hamid Ferdosi, Elisabeth K. Dissen, Ji Li, Jaeil Ahn

**Affiliations:** 1 Center for Epidemiology and Environmental Health, Consultants in Epidemiology and Occupational Health (CEOH), Washington, DC 20016, USA; Hamid@CEOH.com (H.F.); Elisabeth.Dissen@gmail.com (E.K.D.); 2 Department of Health Policy and Management, School of Public Health, Johns Hopkins University-Bloomberg, Baltimore, MD 21205, USA; 3 Department of Pediatrics, School of Medicine, Georgetown University, Washington, DC 20057, USA; 4 Department of Epidemiology and Biostatistics, School of Public Health, George Washington University-Milken Institute, Washington, DC 20052, USA; 5 Department of Pathology, School of Medicine, Johns Hopkins University, Baltimore, MD 28217, USA; JLi42@JHMI.edu; 6 Department of Biostatistics, Bioinformatics, and Biomathematics, School of Medicine, Georgetown University, Washington, DC 20057, USA; JA1030@Georgetown.edu

**Keywords:** arsenic, lung cancer, drinking water, dose-response, risk analysis

## Abstract

High levels (> 200 µg/L) of inorganic arsenic in drinking water are known to be a cause of human lung cancer, but the evidence at lower levels is uncertain. We have sought the epidemiological studies that have examined the dose-response relationship between arsenic levels in drinking water and the risk of lung cancer over a range that includes both high and low levels of arsenic. Regression analysis, based on six studies identified from an electronic search, examined the relationship between the log of the relative risk and the log of the arsenic exposure over a range of 1–1000 µg/L. The best-fitting continuous meta-regression model was sought and found to be a no-constant linear-quadratic analysis where both the risk and the exposure had been logarithmically transformed. This yielded both a statistically significant positive coefficient for the quadratic term and a statistically significant negative coefficient for the linear term. Sub-analyses by study design yielded results that were similar for both ecological studies and non-ecological studies. Statistically significant X-intercepts consistently found no increased level of risk at approximately 100–150 µg/L arsenic.

## 1. Introduction

Inorganic arsenic in drinking water occurs naturally from the earth’s crust and anthropogenically as a byproduct of some industrial processes and uses. While human arsenic exposure can be air-borne or food-borne (generally organic arsenic), the biggest cancer health threat from inorganic arsenic in the environment is from drinking water [1]. Organic arsenic compounds, which are abundant in seafood, are less harmful to health and are rapidly eliminated by the body [1]. 

Exposure to inorganic arsenic through drinking water causes bladder, skin, and lung cancers and has been classified as being carcinogenic to humans by the International Agency for Research against Cancer [2] and as a known human carcinogen by the U.S. Environmental Protection Agency [3]. Multiple studies around the world have demonstrated the increased risk of bladder and lung cancers with exposure to drinking water containing high levels of inorganic arsenic. The studies from Taiwan [4,5,6] and Chile [7,8,9] have received the most attention. Additional non-USA studies include those from Argentina [10,11], Bangladesh [12] and Japan [13]. Previous systematic reviews of lung cancer and ingested arsenic exposure have pointed out the significant associations between lung cancer and high arsenic exposures [14,15]. 

Lung cancer risk from exposures to arsenic in the hundreds of µg/L range but not below 100 µg/L has been consistently found. The dose-response pattern transitioning from relatively low risk at low exposure levels to high risks at high exposure levels is unknown. The purpose of this analysis is to examine across this low to high concentration range. The dose-response patterns might be evident in the assembly of the data while not otherwise evident in the examination of single studies. Furthermore, we additionally wish to examine whether such patterns are consistent across study designs. 

We have assembled the published literature on lung cancer and drinking water arsenic levels and examined the risk over the spectrum of exposure—from about 1 ppb (1 µg/L) to 1 ppm (1000 µg/L). This range was chosen since some publications have suggested that there is a carcinogenic threshold in the 100 µg/L to 200 µg/L range [16,17]. Such a threshold would be consistent with the more recent toxicological/Mode of Action (MOA) findings of arsenic exposure initially inducing a cellular toxicity that leads secondarily to a cellular proliferation. This proliferation then promotes the growth of induced or endogenous mutations, which subsequently lead to additional cancer [16,18,19]. We shall examine the epidemiological literature to identify the dose-response pattern over this exposure range. 

## 2. Materials and Methods

### 2.1. Source Material 

A search of electronic literature bases (PubMed [N = 92], Embase [N = 165], Web of Science [N = 26], and Scopus [N = 14]) was conducted in August 2015 using the keywords “drinking water arsenic”, “lung cancer”, and “epidemiology”. There was no restriction on geography or on language. This yielded 202 citations from 1981 through 2015. Their titles and/or abstracts were reviewed. Reviews were examined to identify primary studies that might have been missed. Subsequent analyses of previously published studies were also sought. The preliminary search was for studies with risk estimates at various exposure levels including low exposures. The selection was then restricted to studies that reported lung cancer risks in a defined study population of males and females combined, used drinking water arsenic level in units of µg/L as the exposure metric, and had both a reference population with lung cancer at low arsenic exposure as well as additional study populations at both low and high arsenic exposure with high exposure being defined here as greater than 100 µg/L. The most recent data presentations for each study were used in our analyses [20,21]. 

### 2.2. Study Populations 

The search sought ecological, case-control, and cohort studies and was updated on 13 August 2015. There was no restriction on country of study or language of publication. We used the study populations as defined by the authors. All studies had been designed to assess the relationship between drinking water arsenic level and lung cancer incidence or mortality. Incidence cases came either from local and national cancer registries, or hospital records. Mortality cases came from vital statistics records at the national, state, or local level.

### 2.3. Exposure 

All exposure measurements are for arsenic (As) water concentration given in micrograms arsenic per liter (ug As/L) drinking water, equivalent to parts per billion (ppb). Exposures are expressed as either mean or population-weighted mean where feasible; otherwise, mid-range or highest exposures were used. Arsenic exposure data were log-transformed to log arsenic (Log As) as the exposure metric for analyses of individual studies since the exposure range covered three orders of magnitude. In the meta-regression analysis, the exposure metric was transformed to Log [(As − Ref) +1] due to the requirements of the analysis, where As was the arsenic water concentration for the study population in the particular study and Ref was the arsenic water concentration (µg/L) for the reference group in the particular study. 

### 2.4. Outcome Metric 

Studies produced outcomes of lung cancer risk as standardized mortality ratios (SMR) in ecological studies, odds ratios (OR) in case-control studies, and incidence relative risks (RR) in a cohort study. Relative risks or risk ratios were developed for the ecological studies using the SMR in the lowest exposure group as the reference risk and presented as the ratio of the SMR in the specific exposure strata to the SMR in the reference exposure strata. Similarly, the risk ratios for the case-control and cohort studies used the odds ratios or adjusted relative risks with the lowest exposure group as the reference risk. 

### 2.5. Analysis 

For each individual study, the data were displayed on a scatter plot with log risk ratio (Log RR) as the dependent variable and log arsenic (Log As) as the independent variable. A visual examination revealed a non-linear pattern with an upward curve at higher exposure levels. A variety of non-linear models (polynomial through cubic, logistic, exponential, and power models) were examined for their fits to the data with the most consistent pattern seen for a linear-quadratic model. The AIC goodness-of-fit was used to assist in model selection. Each study population was examined for a goodness-of-fit to a linear-quadratic regression model performed in Microsoft Excel [22] with a *p* (*p*-value) for the model at a two-tailed significance level of 5%. Log Arsenic is retained as the expression of exposure rather than Arsenic, as the AIC goodness-of-fit statistic in the all studies model was smaller when Log Arsenic was used (−0.468) than when Arsenic was used (−0.248). 

For a pooled regression analysis, the data from all studies were similarly displayed. A linear-quadratic regression model was performed utilizing each data point from each study population in the analysis for all studies. For the pooled regression analyses by study design, the data were limited to those of the studies meeting that specific study design. Log of the risk ratio (Log RR) was the dependent variable, and log arsenic (Log As) was the independent variable in the regressions performed, yielding a set of coefficients with a *p*-value for each of the coefficients where a two-tailed significance level is set at 5%.

Models were examined for their statistical significance (Prob > F values) and for the coefficients of the linear and quadratic terms with their statistical significance. Where the constant term was not statistically significant, the regressions were run in a no-constant model. 

For the meta-regression analyses, the data were organized by study and analyzed using a linear-quadratic meta-regression model as a series of subsets of data, one set for each study. Meta-regression analyses were conducted using the “metareg” function in STATA, 13.2 [23]. The data points belonging to a specific study population were not themselves independent but did comprise separate data sets that were analyzed as groups. The uncertainty in the risk estimate of each data point was essentially a reflection of its number of cases and those in the reference point for that study population. Analyses were conducted for all studies together and for each study design.

The independent (exposure) variable was transformed to log [(As − Ref) + 1] in order to examine the pattern of the change in risk as the exposure level increased above the reference exposure level. The (As − Ref) in the transformation accommodates the differences in reference levels among the studies and provided a common starting point, and the (+1) in the transformation accommodated for the exposure variable being a logarithmic function and for the fact that log (zero) would be undefined. The net effect of the transformation was that each model began at the starting point of no increased lung cancer risk at no increased arsenic exposure, *i.e.*, not above the reference level. Thus, at arsenic (As − Ref) = 0 µg/L, log [(As − Ref) + 1] is log (1) or zero on the logarithmic scale. The reference point waszeroed out, and, when the constant term in the regression was not significant, a no-constant model was run. 

We conducted a random effects meta-regression model that addressed these aspects, written as

$$Eq.\;1\;\;\;\;\;\;\;\;\;\;{\text{Log RR}}_{i}=\beta_{1}LA_{i}^{2}+\beta_{2}LA_{i}+\;\mu_{i}+\epsilon_{i},\text{ where }\mu_{i}\sim N\left(0,\;\tau^{2}\right)\text{ and }\epsilon_{i}\sim N\left(0,\;\sigma_{i}^{2}\right),$$

where ${\text{Log RR}}_{i}\;$is log-relative risk, and ${\text{LA}}_{i}\;$is log [(arsenic − reference) +1] level in study i, respectively. The random effect$\;\mu_{i}$ is the study *i*th specific randomness that varies between studies. Here $\tau^{2}$ accounts for between-study variance, where a smaller value of $\tau^{2}\;$implies more homogeneous outcome-covariates associations across studies. The quadratic term is retained according to the smaller AIC goodness-of-fit statistics (−0.466) compared to that of the linear model (−0.295) in all types of studies. The inclusion of a cubic term did not improve the goodness-of-fit. *p*-values for the coefficients of the linear and quadratic term coefficients were obtained, as were *p*-values (*p* > F) from the joint test of the null hypothesis, *i.e.*, that the coefficients of $\beta_{1}$ and $\beta_{2}$ are equal to zero after the Knapp-Hartung modification of standard errors [24]. The heterogeneity measures $I_{res}^{2}$ and between study variance $\tau^{2}$ were also computed [25]. Meta-regression was performed using “metareg” function in STATA. For each model, where the linear coefficient was negative, and where the quadratic coefficient was positive, the X_intercept_, the point at which increased risk is zero (*i.e.*, Relative Risk = 1.0), was calculated as the antilog of the ratio of the coefficients (−linear/quadratic) and is quite similar to the no-increase risk level for the data.

## 3. Results

### 3.1. Literature Search

The initial search identified 20 studies for consideration, six of which met the specific study parameters. Four studies were excluded because the exposure media were not drinking water arsenic levels—soil or sediment [26], toenails [27], urine [28], or air [29]. Three studies were excluded because the drinking water arsenic metric was not expressed as µg/L—cumulative exposure [30] or well water arsenic level distributions [31,32]. Two studies were excluded because of outcome measures—no lung cancer in reference population [12] or outcome was a slope rather that a risk ratio [33]. Four studies were excluded because their highest exposure level was in the low exposure range at 10 µg/L [34], 7.58 µg/L [35], 26.5 µg/L [36], and 60 µg/L [37]. One study was excluded because information was incomplete with different exposure intervals for males and for females and no case or control information for non-smoking females [12]. Finally, the Argentinian lung cancer study was excluded because of exposure uncertainty, as its well water measurements were from the 1930s when the assay had a limit of detection of 40 µg/L [11]. In contrast, the other studies used a later assay with a limit of detection of 10 µg/L or lower. A total of six studies met the inclusion criteria.

### 3.2. Study Populations

These 6 studies included three studies from Chile [20,38,39], two studies from Taiwan [21,40], and one study from the United States [41]. These consisted of two ecological studies [38,40], three case-control studies [20,39,41], and one cohort study [21]. The two ecological studies were mortality studies with standardized mortality ratios (SMRs) as the expression of risk. The three case-control studies were incidence studies with odds ratios (ORs) or adjusted odds ratios as the expression of risk, and the single cohort study was an incidence study with adjusted relative risks.

The data from the six studies were analyzed as six study populations with males and females combined. Ferreccio *et al.* (2006) [38] reported analyses for four locations and for two time periods (1985–1992 and 1993–2002). The data for the two contiguous time periods were combined into a single data set for each location for the purposes of this analysis. All of the studies were restricted to adults, defined here as ranging from ≥ 20 year-old(Morales *et al.*, 2000) [40] to ≥ 40 year-old (Bogen *et al.*, 2014) [21] with the exception of Ferreccio *et al.* (2006) [38], which appears to be for the full population. Since lung cancer prior to age 20 is a rare event, it is unlikely to affect the analysis. With the exception of Morales *et al.* (2000) [40], the studies did not provide the opportunity to analyze male and female data separately. The critical information for each of these studies in the analysis is in Table 1 and organized by study design. Details of the individual studies and the derivations of their exposure estimates and risk ratios are presented in Appendix A. Each of these studies has data from three or more exposure levels, as recommended by the National Research Council (2013) [42] for dose-response analyses for inorganic arsenic.

**Table 1:** Studies with characteristics and data.

Author		Location		Period		Outcome		Exposure Metric		Exposure (µg/L)	Relative Risk
Ecological Studies											
Ferreccio 2006		Chile		1985–2002		Mortality		Highest		<10	1.00 (ref)
										287	0.93
										636	3.51
										860	3.08
Morales 2000		Southwest Taiwan		1973–1986		Mortality		Pop-Wt. Mean		33.1	1.00 (ref)
										66.3	0.82
										115.2	0.91
										246.3	2.28
										336.3	1.3
										446.7	2.4
										524.4	2.03
										693.9	3.11
Non-Ecological Studies											
Case-Control Studies											
Dauphine 2013		California & Nevada, USA		2002–2005		Incidence		Mid-Range; Mean		≤10	1.00 (ref)
										42.5	0.75
										173	0.84
Ferreccio 2013		Chile		2007–2010		Incidence		Pop-Wt. Mean		10.2	1.00 (ref)
										60	0.77
										377.5	1.38
										860	2.39
Smith 2009 (Ferreccio 2000)		Chile		1994–1996		Incidence		Pop-Wt. Mean		1	1.00 (ref)
										12.8	0.7
										154	3.4
										636	4.7
										600	5.7
										860	7.1
Cohort Study											
Bogen 2014 (Chen 2010)		Northeast Taiwan		1991–1994		Incidence		Mean		1	1.00 (ref)
										3.26	0.57
										25.9	0.73
										74.3	0.68
										160	1.08
										711	1.57

### 3.3. Exposure Metrics 

The arsenic exposure levels for each of the studies are expressed in µg/L (ppb) and for the most part presented in the published studies as exposure ranges [41], mean [21], or highest exposure [38]. Population-weighted means were calculated where population numbers for strata within the exposure ranges were given [20,39,40]. In the Chilean studies, each area was assigned its highest known arsenic level: Antofagasta and Meijillons (860 µg/L). Tocopilla and Maria Elena (636 µg/L rather than 250 µg/L), and Calama (287 µg/L rather than 150 µg/L), were analogous to that used in Ferreccio *et al.* (2013) [39]. The exposure data for the Southwest Taiwan ecological study, from which mean village well water arsenic levels were developed, were published by the National Research Council (1999) [43].

Smoking is the only other significant exposure for lung cancer. Three studies provided smoking-adjusted results across the arsenic exposure spectrum [20,21,41], and one study had information for only the extremes of the arsenic exposure spectrum [39]. No information on smoking was given in ecological studies [38,40]. None of the studies provided data that enabled distinguishing between risks in smokers and risks in non-smokers. 

### 3.4. Outcome

Lung cancer cases were identified by the original authors from national or local cancer registries [21,41], from national or local death registries [38,40], or from physician or hospital contacts [20,39]. Controls for case-control studies were obtained from random digit dialing [41], from random selections from electoral registry [38], or from frequency-matched hospital and cancer patients [20]. 

Relative risks or risk ratios were utilized in the analyses with the lowest exposure group serving as the reference group. Risk ratios for the ecological studies were calculated using the SMRs (standardized mortality ratios) for each exposure strata [20,38]. Risk ratios for the case-control studies used the odds ratios or adjusted odds ratios for each exposure strata [20,39,41]. Risk ratios for the cohort study used the adjusted relative risks for each exposure strata from the study [21]. 

### 3.5. Study Design 

Study designs separate into ecological studies in which information on cases and comparisons are known on a group basis, such as the number of deaths or cases in a certain age interval and time period in geographic areas with known arsenic exposure levels, but without specific information on individual cases. The non-ecological studies—case-control or cohort—are distinct from the ecological studies in that they contain specific information such as smoking history on individual subjects, often obtained by interview. 

### 3.6. Dose-Response Analysis

#### 3.6.1. Single Studies

**Figure 1:**
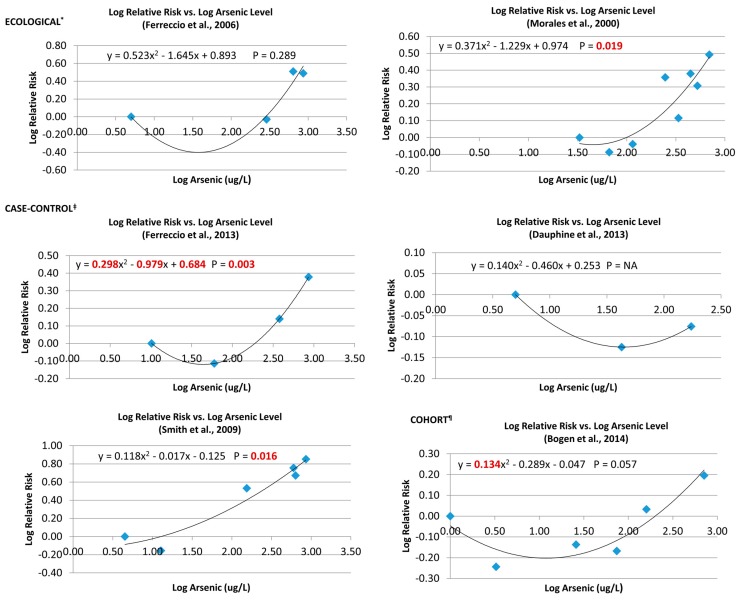
Log-Log plots of individual studies. * Ecological Studies: Ferrecio *et al.*, 2006 [38] (Chile), Morales *et al.*, 2000 [40] (SW Taiwan); **^ǂ^** Case-Control Studies: Ferrecio *et al.*, 2013 [39] (Chile), Dauphine *et al.*, 2013 [41] (USA), Smith *et al.*, 2009 [20] (Chile), Cohort Study: Bogen *et al.*, 2014 [21] (NE Taiwan). Note: log 1 = 0; log 10 = 1; log 100 = 2.

The data from each study have been visually displayed in log RR *vs.* log As scattergrams and analyzed with a linear-quadratic regression model (Figure 1). The data approximate a good visual fit to the model in each of the studies. Three study populations showed a statistically significant goodness of fit (Prob>F value) for the linear-quadratic model. These three were the SW Taiwan ecological study [40] and the two Chilean case-control studies [20,38]. The quadratic term was significant in two analyses [21,39]. The intercept term was significant only in the analysis of the Ferreccio *et al.* (2013) [39] study.

Five of the six studies only presented combined data for males and females together. In the one study for which data for males and females could be analyzed separately, the results for the males (y = 0.37x^2^ – 1.18x + 0.86; *p* = 0.05) and for the females (y = 0.45x^2^ – 1.60x + 1.43; *p* = 0.04) were quite similar to each other and to that with the data combined (y = 0.37x^2^ − 1.23x + 0.97; *p* = 0.02) [40]. 

This circumstance of multiple studies appearing to show a similar regression pattern, although not all with a statistically significantly fit, is an appropriate opportunity for pooled analyses and for meta-regression analyses. 

#### 3.6.2. Pooled Analysis

**Figure 2:**
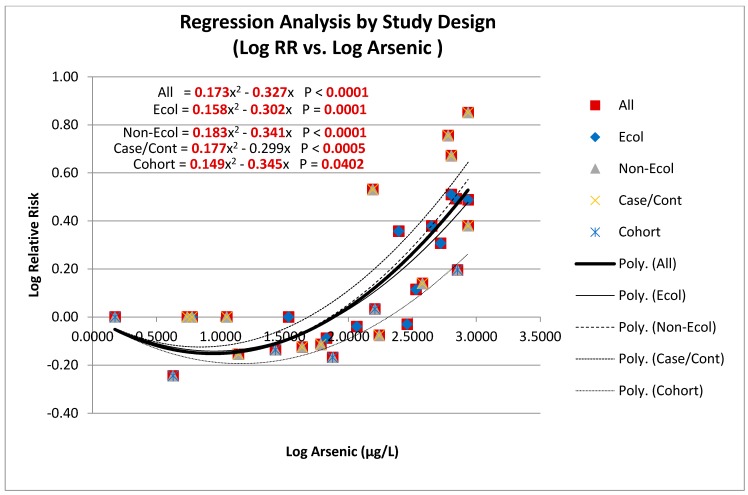
Log relative risk by log arsenic for all study designs.

The study base consists of the studies (N = 6) and their data points (n = 31). Each data point is defined by a log RR value and a log Arsenic value. Figure 2 presents a display of the pooled data from the six studies with linear-quadratic regression models for all the data and for each subset of data organized by study design. The models with their analytic results are shown for the six study data sets and for sub-sets based on study design—Ecological (N = 2; n = 12) and Non-Ecological (N = 4; n = 19); Case-control (N = 3; n = 13) and Cohort (N = 1; n = 6). There is an excellent (*p* < 0.0005) fit in most of the analyses and a good (*p* < 0.04) fit in the cohort analysis. 

The analyses were first run with a constant term. However, as the constant term had been non-significant in each of the analyses, the analyses were subsequently run without the constant term. All regression models show a similar pseudo-parabolic pattern. In each analysis, the quadratic term is positive and always statistically significant, and the linear term is negative and (with one exception) statistically significant. The exception is for the linear term of the analysis of the case-control studies, where the *p*-value was 0.051 rather than < 0.05. 

The regression lines for each of the study design groups display in Figure 2 the same pattern as those of all studies. All have statistically significant positive coefficients for the quadratic term with statistically (or nearly) significant negative coefficients for the linear term. All have a strong outcome-covariate association (*p* < 0.0005) for the model, with the exception of the cohort analysis, which shows a good outcome-covariate association (*p* = 0.04) for the model. Most interesting is that the linear-quadratic regression models for the ecological and non-ecological studies are remarkably similar. The single cohort study yielded a model that is somewhat deeper than the others. 

#### 3.6.3. Meta-Regression

The above analyses have demonstrated the results of pooling the data points within the various studies in order to ascertain the dose-response relationship over the exposure range of observation. Intrinsic to this analytic method are the assumptions that each data point carries the same weight and that each data point is independent of the others. As the data do not fulfill these assumptions, meta-regression analyses (Table 2) have been conducted to address these issues. 

The meta-regression analysis takes into account the variance of each data point by the inclusion of a standard error term based on the number of cases and their comparisons. It further maintains the study source of each data point as a linked set of data.

The desire of an analytic model should include that there is no increased lung cancer risk at zero additional arsenic exposure. As described, we have transformed the exposure measure from log Arsenic to log (As − Ref + 1). The abscissa intends to express the increased exposure above the reference level for each of the study exposure levels. Thus, the adjustment (As − Ref) takes into consideration that different studies have different reference exposure levels and moves each study to the same starting point of no increased exposure above the reference level or zero. However, since the log of zero is undefined, we have transformed the exposure variable from log (As − Ref) to log [(As − Ref) + 1] so that, when (As − Ref) is zero, [(As − Ref) + 1] is 1 and log [(As − Ref) + 1] is zero. Thus, the model begins at no increased lung cancer risk and at no increased arsenic exposure level.

**Table 2:** Meta-regression results by study design.

(As − Ref) +1	N ^o^	β Quadratic	*p*-Quadratic *	β Linear	*p*-Linear *	*Prob* > F	X_intercept_
All	25	0.2139	0.000	−0.4564	0.002	0.000	136.2
Ecological	10	0.2442	0.029	−0.5378	0.065	0.001	159.4
Non-Ecological	15	0.1833	0.013	−0.3796	0.034	0.009	117.7
Case-Control	10	0.1892	0.050	−0.3766	0.113	0.022	97.9
Cohort **^+^**	5	0.1488	0.022	−0.3445	0.023	0.049	206.9

**^O^** Number of data points. * *p*-quadratic and *p*-linear refer to the *p*-values of the quadratic and linear terms, respectively. **^+^** The cohort results are from a simple regression rather than a meta-regression, as there is only one cohort study.

The meta-regression analyses (Eq. 1) reveal that the data, after controlling for study population and including the uncertainty estimate of the log RR [SE (Log RR)], yield an excellent fit (*p* > F) to the no-constant linear-quadratic regression model for all study populations, for study populations from ecological studies, and for study populations from non-ecological studies. Additionally, the data yield a good fit for study populations from the case-control studies and from the cohort study. The regression for the cohort study is a simple no-constant linear-quadratic regression model, as a random effect analysis, would have no meaning for a single study. 

The primary model, which included all study populations and all study designs, yielded a quadratic term (~0.21) with a statistically significant positive coefficient and a linear term (~0.46) with a statistically significant negative coefficient. Sub-analyses were conducted by study design. The sub-analyses for study populations in ecological studies and for study populations in non-ecological studies yielded similar results, having similar positive statistically significant coefficients for the quadratic term (0.21 · 0.03) coefficient, similar negative coefficients for the linear terms (~0.45 · 0.08), and an excellent goodness-of-fit (*Prob* > F value) of < 0.01. Both the analysis of the study populations in the case-control studies and the study population in the cohort study had quadratic terms with statistically significant positive (~0.17 · 0.02) coefficients, linear terms with negative (~−0.365 · 0.016) coefficients that was statistically significant only for the cohort study, and a good goodness-of-fit (*Prob* > F value) of < 0.05. 

**Figure 3:**
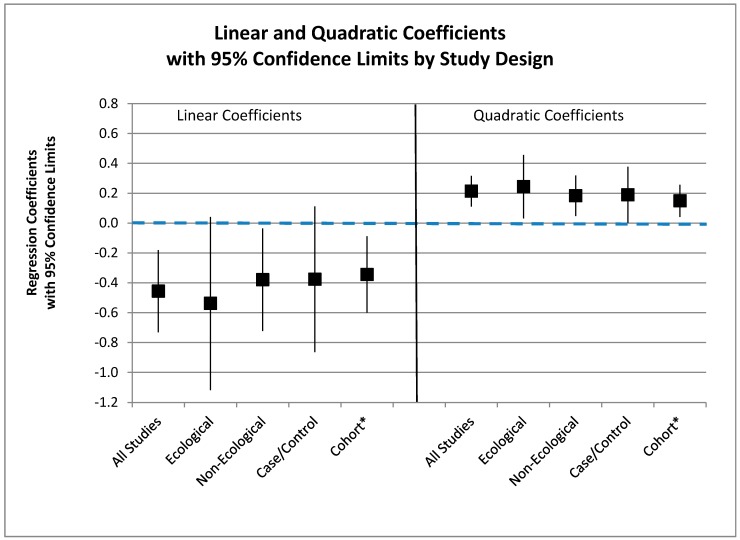
Linear and quadratic coefficients by study design. * Regression coefficients; others meta-regression.

All five models have negative coefficients for the linear term, which, for the meta-regression analysis, for all studies, for the regression and for the cohort study, are statistically significant. There was more variation (−0.34 to −0.18) in the coefficients for the linear term than for the coefficients for the quadratic terms. The study heterogeneity measure $I_{res}^{2}$ accounts for the residual variation due to heterogeneity not explained by the outcome-covariate associations; it was small (37%) for the analysis of the ecological studies and was 0% for the other meta-regression analyses (Not shown). The estimated ${\hat{\tau }}^{2}$ indicates the magnitude of the between study variance and was also small—0.009 for the all-study analysis, 0.014 for the ecological analysis, and zero for the non-ecological and case-control analyses [Not shown]. The coefficients from the meta-regressions and the cohort regression and their 95% confidence intervals are displayed in Figure 3.

The linear coefficients are all seen to be negative and similar, with three out of five being statistically significantly negative, and the quadratic coefficients are all seen to be positive and similar, with five out of five being statistically significantly positive. Thus, the standard pattern is an excellent fit to a no-constant linear-quadratic model with a statistically significant positive coefficient for the quadratic term and a variably statistically significant negative coefficient for the linear term. 

#### 3.6.4. Zero X-Intercept 

The X_intercepts_ of the models, *i.e.*, the non-zero increased arsenic exposure level at which Log RR = zero and RR = 1.0, were obtained. Overall, the all-study populations analysis yielded a X_intercept_ of 136 µg/L. The X_intercept_ for the ecological studies was 159 µg/L, while that for the non-ecological studies was 118 µg/L. The X_intercept_ for the case-control studies was 98 µg/L, while that for the cohort study was 207 µg/L. Each X_intercept_ was statistically significant with its 95% CI not including zero. The interval between 0 and X_intercept_ can be interpreted as approximately the range of an increased level of arsenic exposure for which the model would not predict an increase in the risk of lung cancer. 

The data from the studies on arsenic ingestion and lung cancer, grouped by study design, are each found to fit similar no-constant linear-quadratic log-log meta-regression models. All the meta-regressions show an excellent (*p* < 0.01) fit to the model with statistically significant positive coefficients for the quadratic term. The coefficient for the linear term is negative in each model and is statistically significant in the overall model. The regression for the cohort study shows a good (*p* < 0.05) fit to the model with a statistically significant positive coefficient for the quadratic term and also a statistically significant negative coefficient for the linear term. The coefficient for the quadratic term is similar in all the analyses, and the coefficient for the linear term in the analysis of the cohort study is more greatly negative than that of the others. The regression results based on the ecological studies are not different from the results based on the non-ecological studies.

In summary, regression analysis using a no-constant linear-quadratic model with log-transformed data, both for a pooling analysis and for a meta-regression, finds a general pattern of excellent fit to the model with a mostly statistically significant negative linear term, an always statistical significant positive quadratic term, and a statistically significant X_intercept_ generally in the 100 µg/L to 150 µg/L range with an overall X_intercept_ of 136 µg/L.

## 4. Discussion

We have reviewed the literature on lung cancer risk and drinking water arsenic exposure and have performed a set of regression analyses of the data found. We found six studies whose study population met the study inclusion criteria. As neither the lung cancer risks nor the arsenic concentrations as a group distributed normally, we performed our analyses applying the log-transformation to make data normally distributed. A variety of models were examined, and the data were found to best fit a no-constant linear-quadratic model. Simple regression and meta-regression analyses were conducted with the data for all study populations and separated by study design—ecological studies and non-ecological studies, followed by case-control studies and the cohort study. The ecological studies were mortality studies, and the non-ecological studies were incidence studies. We found that the fitted models for each group of studies gave similar results. In general, the goodness-of-fit for the models was good to excellent, with coefficients for the quadratic terms that were all statistically significant and positive, and coefficients for the linear term that were uniformly negative and quite frequently statistically significant. Further, each model leads to the identification of an exposure level (generally about 100–150 µg/L), below which there is, with a range of uncertainty, no evidence of an increased risk of lung cancer, nor is there evidence of a negative, or hermetic (J-shaped), response.

Other systematic reviews have excluded ecological studies from their meta-analyses on the basis that non-ecological (case-control and cohort) studies have stronger or more efficient study designs as they have individualized data on exposures and confounders [44,45]. In contrast, we have analyzed the literature for both ecological studies and non-ecological studies. We found that the patterns of the regression models were the same for both ecological and non-ecological, and that the results of their meta-regressions were virtually identical. We conclude that the weight of evidence provided by the ecological studies and by the non-ecological studies are equivalent. 

The definition of the study populations, the exposure strata, and the risk estimates generally followed those of the authors. The exception is that the villages of the Morales *et al.* (2000) study group entered the analyses based on the mean village well water arsenic level rather than the median [40]. The Morales *et al.* (2000) study was the only one to have a reference exposure that was not approximately 1–10 µg/L [40]. The all study meta-regression was rerun with the exclusion of that study and yielded a similar pattern with a X_intercept_ of 153 µg/L. 

As a sensitivity analysis, the meta-regression was run with a variety of inclusion assumptions and found to yield X_intercepts_ that ranged between 100 and 165 µg/L. These alternative analyses included the data from their first publication instead of the data from the most recent presentation when individual studies were either dropped or used the data from their first publication instead of the data from the most recent presentation, and when studies were grouped by geographical area, data source (death certificate *vs.* medical record/registry), presumed nutritional status, or highest exposure. The X_intercept_, when including studies, were limited to those with exposure > 500 µg/L was 135.6 µg/L. 

### 4.1. Biological Plausibility 

Whether arsenic is a “threshold” carcinogen or not has been debated for decades. The U.S. EPA held a roundtable on this topic in 1995 and reached no conclusion [46]. Later, the U.S. Food and Drug Administration (U.S. FDA) led a symposium seeking to “explain the apparent non-linear threshold response for arsenic carcinogenesis” [47]. It was concluded that most modes of action (MOAs) identified for arsenic carcinogenesis supported a non-linear dose-response at low-level exposures. Subsequently, studies have focused on mode of action. 

Snow *et al.* (2005) [19] focused on oxidative stress as mode of action but also considered cellular proliferation or telomerase activation, suggesting a sub-linear dose-response curve]. Schmeisser *et al.* (2013) [48] focused on mitochondrial transcription factors, a process known as mitohormesis. Finally, Cohen *et al.* (2013) [16] provided evidence that the mode of action involved reactive trivalent metabolites interacting with critical cellular sulfhydryl groups, leading to cytotoxicity and regenerative cell proliferation. They concluded that the cytotoxicity induced by inorganic arsenic resulted in non-cancer toxicities, and that the regenerative cell proliferation enhanced development of epithelial cancers, including those of the lung.

The linear-quadratic models demonstrated here are not inconsistent with current thinking on these nonlinear MOA processes underlying arsenic carcinogenicity. 

### 4.2. Risk Modeling

There are some limitations with our linear-quadratic model. It was based on the assumption that a single analytic model would express an exposure-outcome relationship in a continuous model across the full exposure range. It may be that a segmental model with two segments would fit the data better, since the biological model proposed (cellular toxicity followed by cellular proliferation) would not necessitate the same process or dose-response pattern in both exposure ranges. Further analyses could include the extension of a hockey-stick model or a step function in the meta-regression framework with a robust estimation of such a hooked pattern using non-parametric regression approaches. In fact, US EPA (2005) cancer guidelines specifically encourage investigators to contemplate dual modes of action in any dose response assessment when sufficient data on MOA and tumor observations are available [49]. Unfortunately, we are unable to distinguish between these models but propose the continuous model as being most parsimonious. Our judgment here is similar to that proposed by Dourson *et al.* (2008) [50] for the dose-response assessment for acrylamide’s tumorigenicity based on a dual mode of action (MOA), and for similar reasons. 

An alternative interpretation of our model might be that the ingestion of inorganic arsenic stimulates both anti-carcinogenic responses (linear and negative) and pro-carcinogenic responses (quadratic and positive). At some exposure levels, the pro-carcinogenic responses would be greater than the anti-carcinogenic response, leading to the observed effects. Additional efforts would need to be made to explore this speculation and seek a mechanistic basis. 

### 4.3. Limitations

These analyses are able to assess the overarching hypothesis on the shape of the dose-response curve for lung cancer over the range of low to high water arsenic concentration exposures. They are not able to distinguish difference in risk or risk pattern for males *vs.* females or for smokers *vs.* non-smokers. Other sources of arsenic exposure, such as dietary or occupational, have not been included. These analyses intrinsically have assumed that within each study the daily water intake is independent of the arsenic water concentration, though the population water consumption may differ between studies. Most of these study populations have been nutritionally sufficient, and issues of nutritional deficiency and arsenic metabolism are also not included. As it is based on the studies that cover the full exposure range, it has not included those studies that are limited to the low exposure range. Further, these analyses apply only to the carcinogenic risk pattern for lung cancer and not for other adverse effects of inorganic arsenic ingestion, such as cardiovascular, endocrine/metabolic, and reproductive aspects. The carcinogenic risk pattern for bladder cancer has been assessed elsewhere (Tsuji *et al.*, 2014) [45].

## 5. Conclusions

We have conducted a systematic review of the epidemiological literature on drinking water arsenic levels and lung cancer and an analysis of their data. We examined the fit to a wide range of continuous models and found overall, and by study design, that the lung cancer data best fit a linear-quadratic model with a statistically significant positive quadratic coefficient and a negative linear coefficient that was statistically significant in the overall model. Importantly, we found that an analysis of the data from the ecological studies was as informative and with similar results as an analysis of the data from the non-ecological studies (*i.e.*, case-control and cohort studies). All analyses yielded statistically significant X_intercepts_ that generally indicate no increased risk at exposures below about 100–150 µg/L for lung cancer over the low through high arsenic exposure range.

## **Appendix**

### Description of Studies and Available Data

Summaries of the studies’ contributing data to the systematic review follow with a presentation of their data options for exposure and for risk of outcome. The exposure data in bold indicate the exposure data used in the systematic review. The risk statements include 95% confidence intervals when available. Case/Cont indicates the number of cases and controls within the specific stratum in the case-control studies.

### Ecological Studies

Ferreccio *et al.* (2006) [38] reviewed studies on the health impact of arsenic exposure in Chile. With regard to lung cancer, they reported the SMRs for 1985–1992 and 1993–2002 for four cities with different levels of arsenic exposure. The authors presented the average exposures for each city from 1958 to 1970, though for some cities the highest exposures were from 1971 to 1977. They presented SMRs for males and females combined, based on Chilean national data. Our analyses were based on the higher of the 1958–1970 and 1971–1977 exposures.

**City**	Arsenic				SMR	RR			
	**1958–1970**	**1971–1977**	**Highest**	**1985–1992**	**1993–2002**	**1985–2002**	**1985–1992**	**1993–2002**	**1985–2002**
Valparaiso	<10	<10	<10	136.6	131.8	133.9	1.00	1.00	1.00
Calama	150	287	287	140.7	112.5	125.0	1.03	0.85	0.93
Tocopilla	250	636	636	479.1	497.2	433.6	3.51	3.01	3.51
Antofagasta	860	110	860	420.1	406.1	412.3	3.08	3.08	3.08

Morales *et al.* (2000) [40] is an analysis of the 42 village study population in the arsenic endemic region of southwest Taiwan (Wu *et al.* 1989) [5]. The regional data served as the reference population. Drinking water samples were taken from 1964 to 1966 from extant wells in 42 villages. Cancer mortality data for 1973–1986 came from death certificates. Wu *et al.* (1989) [5] had reported a stratified analysis based on median village well water arsenic levels in three strata (<300, 300–600, and > 600 µg/L), and Morales *et al.* (2000) [40] reported the same for adults in eight strata (0–50, 50–100, 100–200, 200–300, 300–400, 400–500, 500–600, and 600+ µg/L). NRC (1999) [43] published a table of well water levels and cancer counts for each village among adults aged 20 y/o or older. Those data revealed that the use of the median led to misclassification errors as some villages with low level medians (10–126 µg/L) had very high exposures (580–770 µg/L) that were not reflected in the medians. We have used the arsenic strata of Morales *et al.* (2000) [40] but have entered the villages on the basis of their means rather than their medians. Within each stratum, we have given both the median mean and the population-weighted mean and have used the population-weighted means for each strata.

**Arsenic**	**Mid-Range**	**Median Mean**	**Pop-Wt Mean**	**SMR**	**RR**
0–50	25	32	33.1	1.80 (1.11–2.75)	1.00
50–100	75	62.5	66.3	1.47 (0.97–2.14)	0.82
100–200	150	122.5	115.2	1.64 (0.82–2.94)	0.91
200–300	250	245.9	246.3	4.10 (3.01–5.44)	2.28
300–400	350	328.5	336.3	2.34 (1.21–3.84)	1.30
400–500	450	448	446.7	4.31 (2.67–6.59)	2.40
500–600	550	529	524.4	3.65 (2.53–5.10)	2.03
600–818	767	665.2	693.9	5.59 (4.51–6.84)	3.11

### Case-control Studies

Dauphine *et al.* (2013) [41] studied histologically-confirmed lung cancer cases diagnosed in 2002–2005 and controls from six counties in western Nevada and one (Kings County) in central California. Over 7000 arsenic measurements from public drinking water sources and private wells were obtained from the state health departments. Arsenic levels have been shown to be relatively stable over time in the area. If a measurement for a well was unavailable, the median of all wells within the same square-mile had been used. Individuals were stratified by their highest 5-year average arsenic exposure after a 40 year lag into ≤10 µg/L, 11–84 µg/L, and ≥85 µg/L. Exposure data for 30 of the 54 in the high group were reported as a mean of 173 µg/L, a median of 110 µg/L, and a mode (24/30) of 110 µg/L. Lung cancer cases (N = 196) were identified directly from hospitals. Cases had to be >25 years old, living in the study area at the time of diagnosis, and able to provide interview data. Controls (N = 359) were selected by random digit dialing and matched by age, gender, and state but not county. Adjusted and unadjusted odds ratios differed little. Mid-range levels were used in the analyses for the lower strata, and the mode was used for the high exposure group.

**Arsenic**	**Mid-Range**	**Median**	**Mean**	**Case-control**	**OR**	**Adj OR**
≤10	**5**			141/241	1.00	**1.00**
11–84	**42.5**			37/82	0.77	**0.75**
≥85	772.5	110	**173**	18/36	0.85	**0.84**

Ferreccio *et al.* (2013) [39] studied lung cancer cases diagnosed between 2007 and 2010 and controls from the 922,579-person population of Regions I and II of northern Chile. Arsenic measurements were available for >97 % of all drinking water sources in the area. Exposure levels were determined for each city and town and presented for seven time intervals between 1930 and 1995 (+). The exposure levels ranged from a high of 860 µg/L for Antofagasta and Mejillones between the years 1958 and 1970 to a low of 10 µg/L or less in Arica and Putre. The authors stratified the exposure into four groups, based on the four largest cities in the study area: Arica (10 µg/L), Iquique (60 µg/L), Calama (150 µg/L in 1958–1970; 287 µg/L in 1971–1977), and Antofagasta (860 µg/L in 1958–1970; 110 µg/L in 1971–1977). Each individual’s exposure was defined as their highest single-year exposure throughout the subject's lifetime, from birth to diagnosis or entry into study. Cases (N = 306) were obtained from pathologists, hospitals, and radiologists. Inclusion criteria for cases were lung cancer diagnosis from 2007 to 2010, resident in the study area, >25 years old, and provided interview data. Controls (N = 640) were randomly selected from electoral registries and matched by age and gender. The authors used as the exposure metric the highest level for the largest city in the strata, which was reasonable for three of the four strata. For the third stratum (200 µg/L–799 µg/L), the highest level in the largest city (Calama) was 287 µg/L, while the highest levels in the other cities in that stratum were 600 µg/L and 636 µg/L. The other cities contained 26% of the population of that stratum. The population-weighted mean for the third stratum was 377.5 µg/L, which we used in our analysis.

**Arsenic**	**Mid-Range**	**Largest City**	**Pop-Wt Mean**	**Case/Cont**	**OR**
0–59	30	10	**10.2**	48/138	**1.00**
60–199	130	60	**60**	52/193	**0.77**
200–799	500	287	**377.5**	69/144	**1.38**
≥800	900	860	**860**	137/165	**2.39**

Smith *et al.* (2009) [20] reported a re-analysis of data from his team’s previously published case-control study (Ferreccio *et al.*, 2000) [51]. This case-control study had compared arsenic exposures of lung cancer cases diagnosed in 1994–1996 and their controls also in regions I, II, III in northern Chile. Ferreccio *et al.* (2000) [51] presented in Table 1 the arsenic exposure for the various towns and cities in the study area for six time intervals between 1930 and 1994. Arsenic concentrations since 1930 have generally remained steady, with the exceptions that two cities (Antofagasta and Mejillones) had a marked increase from 1958 to 1970 and three cities (Tocopilla, Maria Elena, and Calama) had a marked increase from 1971 to 1977. The arsenic concentrations in Antofagasto and Mejillones were 860 µg/L during 1958–1970 and 90 µg/L before and 110 µg/L afterwards. The arsenic concentrations in Tocopilla and Maria Elena were 636 µg/L during 1971–1977 and 250 µg/L before and 110 µg/L afterwards. The arsenic concentrations in Calama were 287 µg/L during 1971–1977 and 150 µg/L before and 110 µg/L after. Smith *et al.* (2009) [20] and Ferreccio *et al.* (2000) [51] analyzed risk in relation to 1958–1970 exposures. We analyzed risk in relation to population-weighted averages using the higher of either the 1958–1970 or 1971–1977 exposure levels.

**Pop-Wt**						
**Arsenic**	**Mid-Range**	**(58–70)**	**(71–77)**	**Highest**	**Case/Cont**	**Adj OR**
0–9	5	1.0	1.0	**1.0**	11/92	**1**
10–59	35	12.8	12.8	**12.8**	7/81	**0.7**
60–199	130	97.3	154	**154**	35/87	**3.4**
200–399	300	250	636	**636**	23/44	**4.7**
400–699	550	600	600	**600**	11/12	**5.7**
700–999	850	860	110	**860**	64/103	**7.1**

### Cohort Study

Bogen *et al.* (2014) [21] presented a re-analysis by C.L. Chen of the Chen *et al.* (2010) [52] study of four townships in the arseniasis-endemic area on the northeastern coast of Taiwan. Residents (n = 8102) had used wells in their own backyards from the 1940s until tap water was implemented in the 1990s. Well water samples collected at the time of interview in 1991–1994 had levels of <0.15 to 3590 µg/L arsenic. Well use histories were obtained. Full exposure histories could be developed for a cohort of 6888 members aged > 40 y/o. Lung cancer cases newly diagnosed from 1991 to 2006 were identified through Taiwan's national cancer registry. The Chen *et al.* (2010) [52] analysis had assigned subjects by their arsenic level at their time of enrollment into four arsenic exposure strata (<10, 10–49.9, 50–99.9, 100–299.9, and ≥ 300 µg/L). Subsequently, Chen released a five-stratum analysis (0–1, 1–<10, 10–<50, 50–<100, 100–<300, and ≥ 300 µg/L) with arithmetic mean arsenic levels and relative risks (Bogen, Chen, and Tsuji, 2014) [21].

**Range**	**Mean**	**Participants**	**Person-Years**	**Cases**	**adj RR**
0–1	1	1071	12,558	32	1.00
1<10	3.26	1371	15,781	23	0.57
10–<50	25.9	2027	23,473	47	0.73
50–<100	74.3	880	9989	19	0.68
100–<300	160	873	10,000	27	1.08
>300	711	666	7525	30	1.57
